# Editorial: Recent advances in vitamin D supplementation for improved reproductive endocrine and metabolic parameters

**DOI:** 10.3389/fendo.2023.1251388

**Published:** 2023-08-18

**Authors:** Faiza Alam, Aysha Habib Khan, Mukhtiar Baig, Rehana Rehman

**Affiliations:** ^1^ PAPRSB Institute of Health Sciences, Universiti Brunei Darussalam, Bandar Seri Bagawan, Brunei; ^2^ Pathology & Laboratory Medicine, Aga Khan University, Karachi, Pakistan; ^3^ Faculty of Medicine, Rabigh, King Abdulaziz University, Jeddah, Saudi Arabia; ^4^ Department of Biological & Biomedical Sciences, Aga Khan University, Karachi, Pakistan

**Keywords:** vitamin D, free vitamin D, reproductive outcomes, endocrine disorders, pregnancy complications

## Introduction

Vitamin D (VitD) deficiency is a global issue that affects all age groups, including women of reproductive age. It has also been associated with a variety of reproductive disorders and adverse pregnancy outcomes. Recent studies highlight the VitD role in regulating male and female reproductive health (1, 2). Matias et al. observed that VitD could reduce preeclampsia-related inflammasome and the TLR4-MyD88-NF-κB pathway activation (3). VitD can also improve insulin sensitivity by increasing insulin receptiveness to glucose transport, reducing the chances of Gestational Diabetes Mellitus. As far as the role of VitD in reproductive physiology is concerned, it was documented to be a predictor of the success of *in vitro* fertilization treatment (4). Supplementation with VitD has been shown to improve pregnancy outcomes (5).

## Published manuscripts

1. A perspective study authored by Liu et al. investigated the influence of thyroid autoimmunity (TAI) and VitD levels on early pregnancy outcomes in women of normal thyroid function who undergo *in vitro* fertilization (IVF). The study found low serum VitD in subjects with TAI with fewer good-quality embryos. Additionally, aging also reduced the chances of pregnancy. The paper provides clinical practice guidelines for managing infertility in patients with normal thyroid function. Future studies should explore the effectiveness of VitD interventions in patients with TAI-assisted reproductive techniques (ART).
2. Another prospective study by Liu et al. emphasizes the influence of TAI on serum and follicular fluid (FF) VitD levels and VitD receptor expression in granulosa cells (GCs) on laboratory results in infertile patients undergoing IVF or intracytoplasmic sperm injection (ICSI) treatment. The study found that TAI has a more detrimental effect on the maturation of good-quality embryos as compared to low VitD levels.
3. A study conducted by Wei-Jiun Li et al.: The original article describes that low VitD levels are a risk factor for Post partum heamorrhage (PPH), and low levels of VitD are associated with an increased likelihood of experiencing low haemoglobin levels before delivery. Therefore, the study recommends that VitD should be corrected during antenatal care to avoid PPH and its complications.
4. Shan et al.: The original article declared that lower serum 25(OH)D levels were associated with higher risks of hyperandrogenemia (HA) in females with polycystic ovary syndrome (PCOS). The study identified increased BMI and age greater than 26 years as risk factors for PCOS women with VitD deficiency which should be considered for HA assessment and correction of VitD.
5. Zhou et al.: This meta-analysis concludes that VitD supplementation improves the chemical pregnancy rate in infertile women with VitD deficiency; however, VitD supplementation does not improve the clinical pregnancy rate and all secondary outcomes. Further studies are needed to explore the role of VitD on other IVF outcomes, like fertilization rate and ongoing pregnancy rates.
6. Another systematic review authored by Li et al. reviews “nine randomized controlled trials (RCTs) for PCOS undergoing therapy with magnesium supplementation alone or in combination with other agents”. The study outcome indicated that magnesium supplementation in combination with other supplements may be a potential therapeutic option for improving metabolic disorders in PCOS patients.

Recent studies have found promising results in using VitD supplementation to improve oxidative stress, reproductive endocrine and metabolic parameters. VitD may positively affect fertility and metabolic health by influencing hormone levels and metabolic markers (6). However, more research is needed to determine the best dosage, duration, and timing for supplementation. Researchers, clinicians, and policymakers must collaborate to establish evidence-based guidelines for using VitD in reproductive health. These advancements in VitD supplementation offer potential benefits for individuals and couples looking to enhance fertility and achieve metabolic balance.

## Author contributions

FA initiated the write-up of the editorial. MB, AK, and RR completed the write-up. All authors revised the manuscript before final submission. All authors contributed to the article and approved the submitted version.
